# Supplementary figures and images for: Fatal Neurodissemination and SARS-CoV-2 Tropism in K18-hACE2 Mice Is Only Partially Dependent on hACE2 Expression
Source: Viruses. 2022 Mar 5;14(3):535. doi: 10.3390/v14030535 (PMC8955233; doi:10.3390/v14030535)

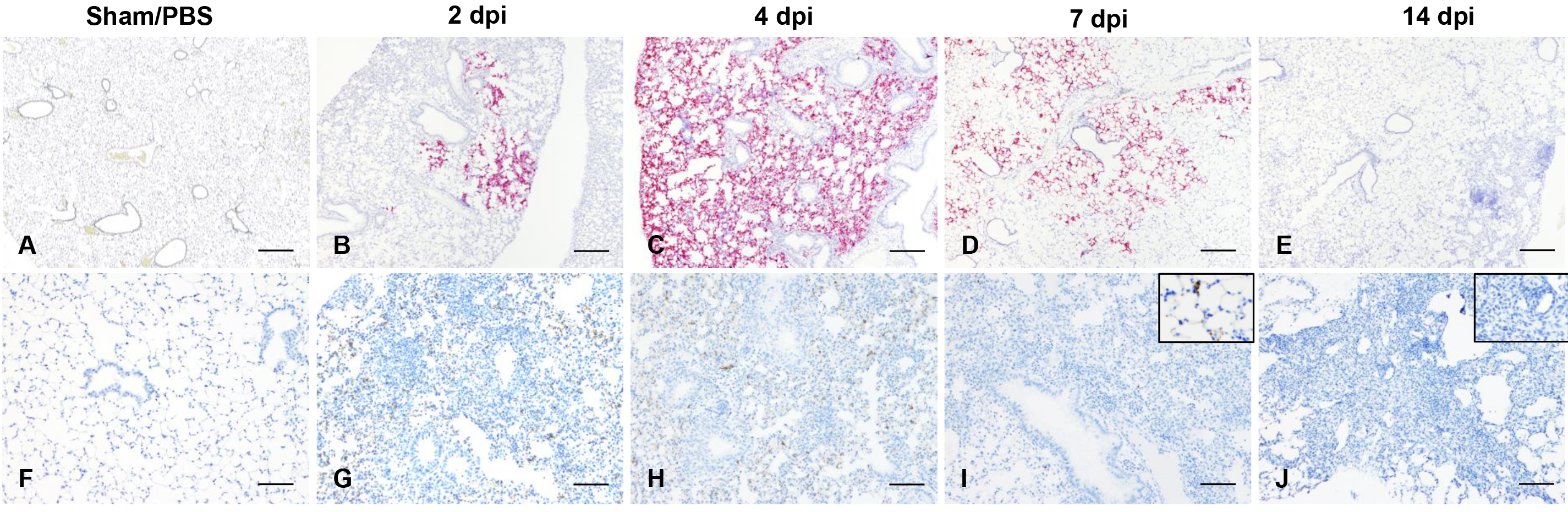

Supplement: Supplementary file 1 [file viruses-14-00535-s001.zip › Figure S1.tif]

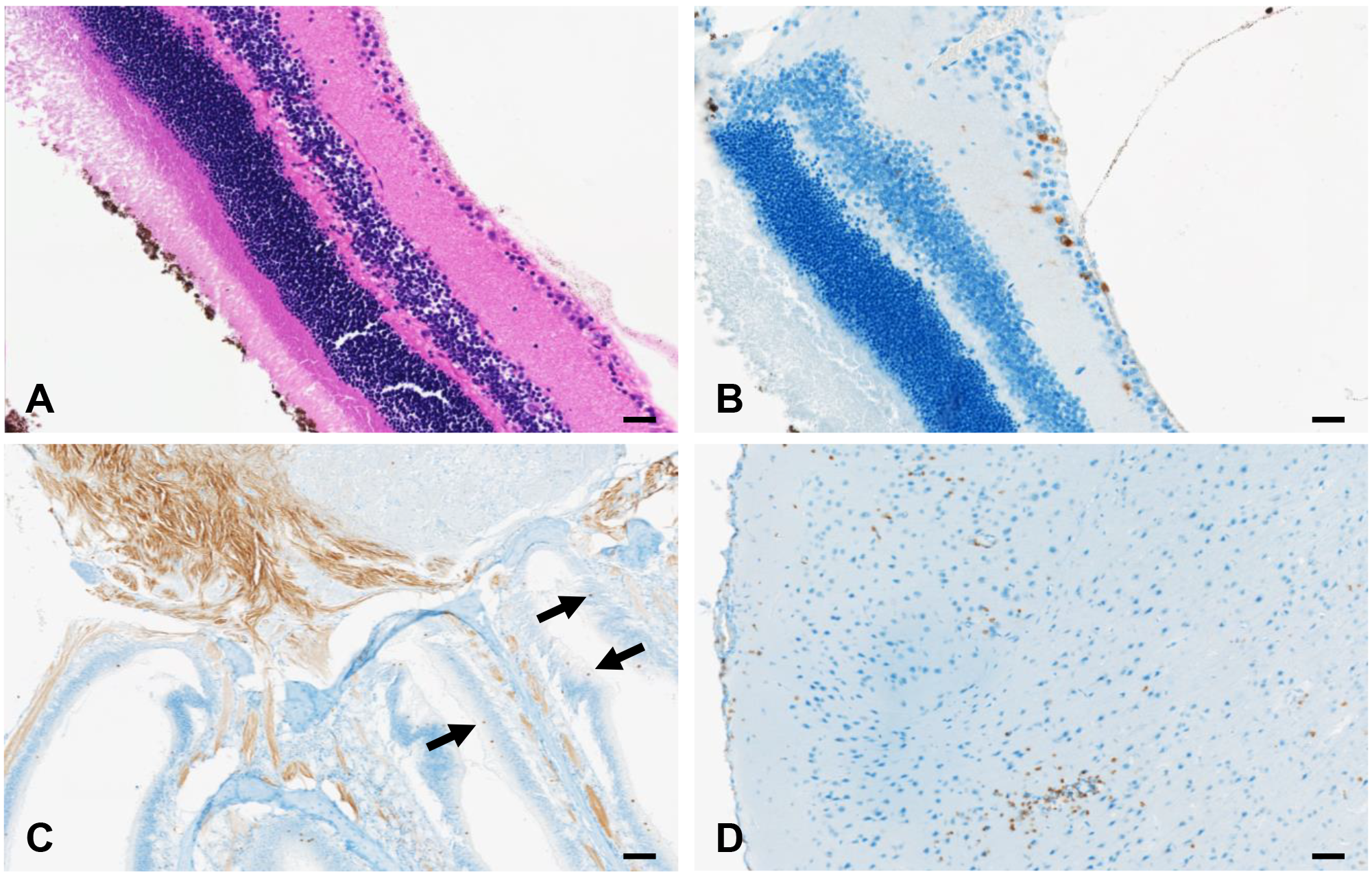

Supplement: Supplementary file 1 [file viruses-14-00535-s001.zip › Figure S2.tif]

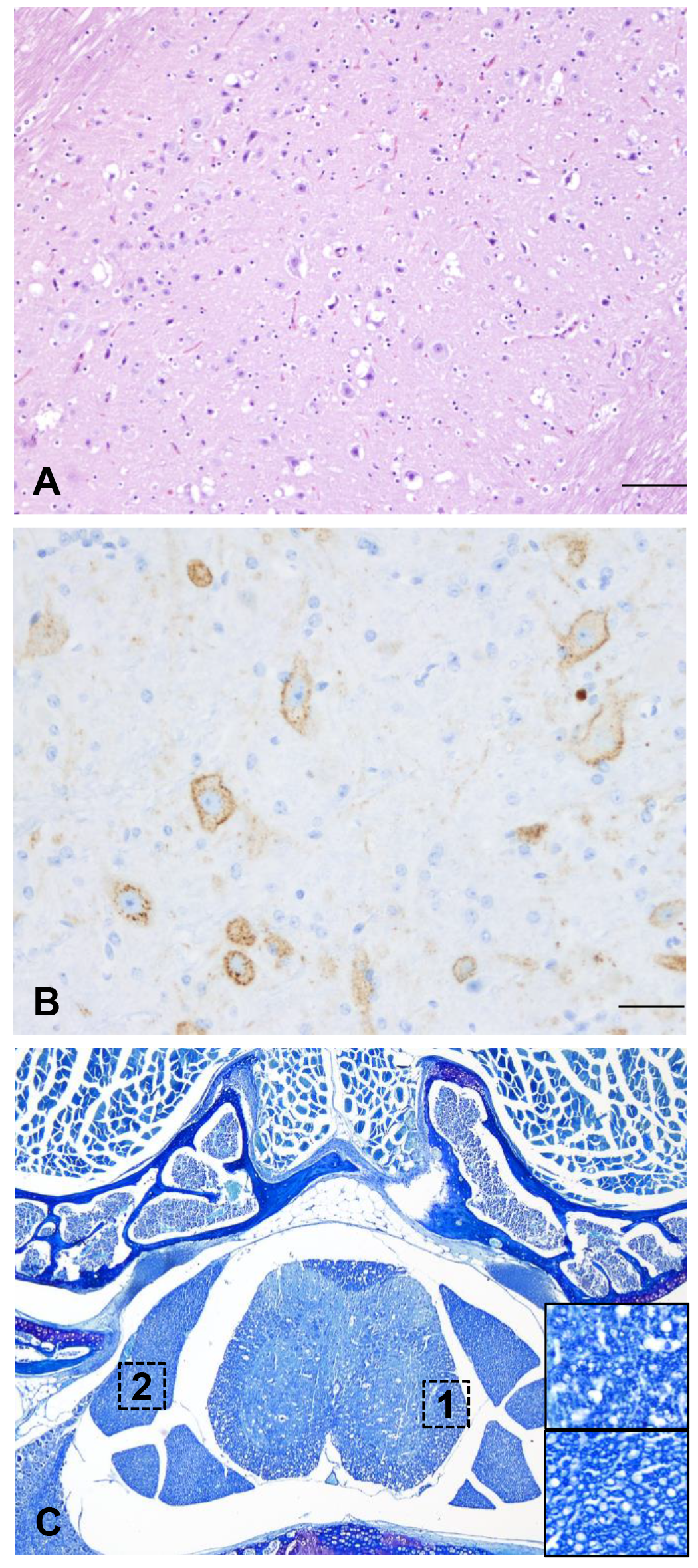

Supplement: Supplementary file 1 [file viruses-14-00535-s001.zip › Figure S3.tif]

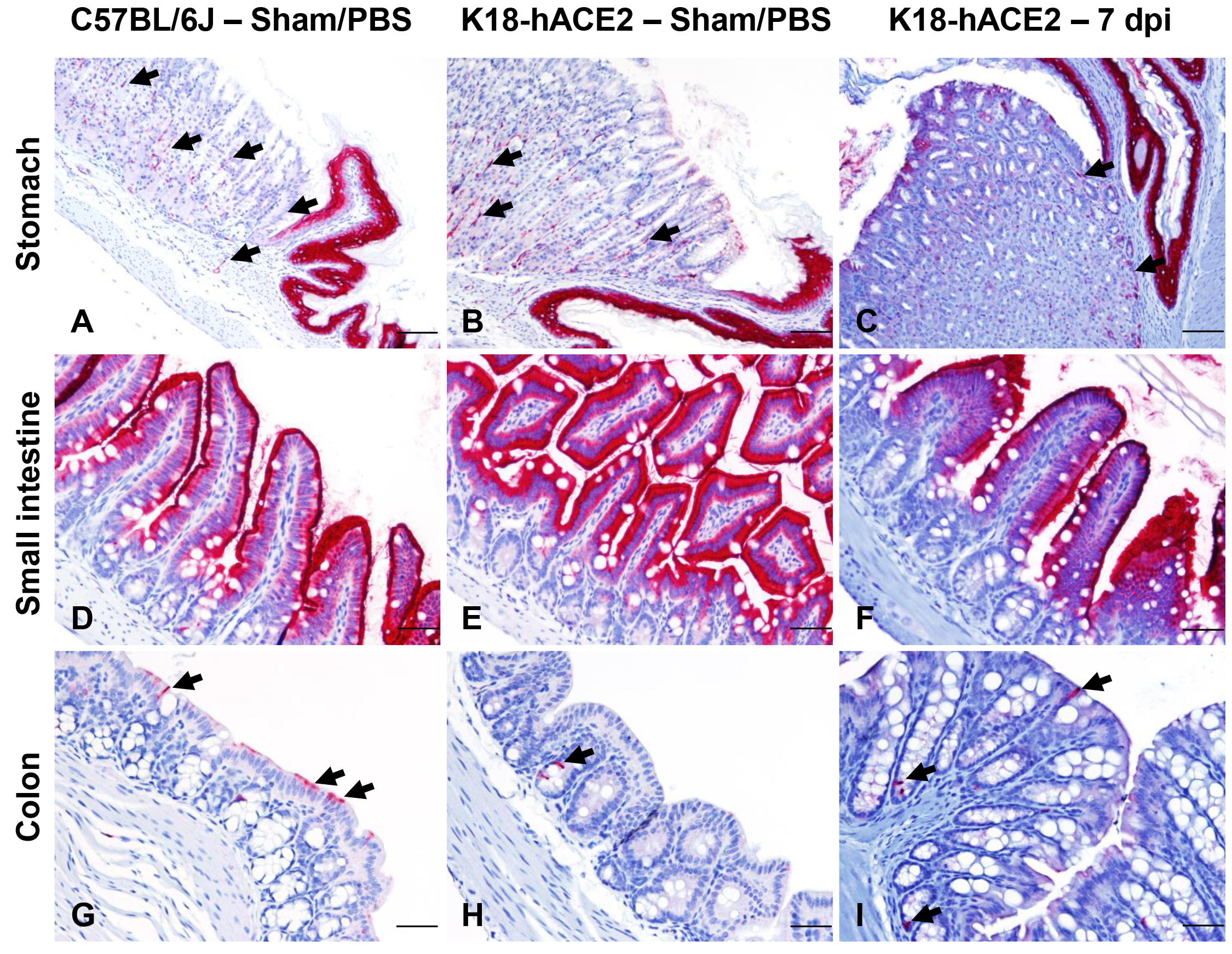

Supplement: Supplementary file 1 [file viruses-14-00535-s001.zip › Figure S4.tif]
